# Better brain connectivity is associated with higher total fat mass and lower visceral adipose tissue in military pilots

**DOI:** 10.1038/s41598-019-57345-3

**Published:** 2020-01-17

**Authors:** David Cárdenas, Iker Madinabeitia, Jesús Vera, Carlos de Teresa, Francisco Alarcón, Raimundo Jiménez, Andrés Catena

**Affiliations:** 1 Department of Physical Education and Sport, Faculty of Sport Sciences, University of Granada, Granada, Spain; 2 Sport and Health University Research Institute (iMUDS), Granada, Spain; 3 Department of Optics, Faculty of Sciences, University of Granada, Granada, Spain; 4 Andalusian Centre of Sports Medicine, Consejería de Turismo y Deporte, Junta de Andalucía, Granada, Spain; 5 Department of Didactic General and Specific Training, Faculty of Education, University of Alicante, Alicante, Spain; 6 Department of Experimental Psychology, Faculty of Psychology, University of Granada, Granada, Spain; 7 Mind, Brain and Behaviour Research Centre (CIMCYC), University of Granada, Granada, Spain

**Keywords:** Cognitive neuroscience, Cognitive neuroscience, Neurology, Neurology

## Abstract

A lack of exercise leads to being overweight or obese affecting regional brain structure and functional connectivity associated with impaired cognitive function and dementia. In recent decades, several studies of healthy individuals suggest that adiposity may also produce negative independent effects on the brain. We aimed to investigate the relationship between body composition – total fat mass (TFM) and visceral adipose tissue (VAT) – with white matter (WM) integrity using a whole-brain approach in military pilots. Twenty-three military helicopter pilots (M_*age*_ = 36.79; SD = 8.00; M_*BMI*_ = 25.48; SD = 2.49) took part in the study. Brain volumes were studied using diffusion tensor imaging technique by means of a 3T Magnetom Tim Trio. Measurements of body mass index (BMI), TFM and VAT were obtained using Dual-energy X-ray Absorptiometry (DXA). The results showed that, on one hand, higher TFM was associated with higher white matter fractional anisotropy (FA) and, on the other hand, higher VAT was associated with lower FA. Data showed that TFM and VAT are the critical factors underlying WM integrity in combat helicopter pilots. The authors suggest that fat presence enhance brain connectivity while there is no excess, specifically in VAT.

## Introduction

Military pilots are exposed to uncertain scenarios in extreme conditions where an incorrect decision may have catastrophic consequences, and thus, the assessment of brain function may help to evaluate the cognitive capabilities of pilots and predict their behaviour in challenging situations^1^. In recent decades, there has been a growing interest in analysing the relationships among body composition, physical fitness and brain function. Several brain imaging studies have shown an association between adiposity and decreased global brain volume^2^, as well as reduced volume of grey matter^3^ (GM) and white matter^4^ (WM). Related to this topic, the accumulated evidence has demonstrated that obesity raises the risk of cognitive decline and dementia. Indeed, it has been associated with structural brain changes in bilateral temporal lobes, anterior lobe of the cerebellum, frontal lobes, temporal lobes, anterior lobe of the cerebellum, occipital lobe, frontal lobe and precuneus^3^ and impaired functional connectivity^5^. Taking these results into account, we suggest that the putative link between functional brain alterations and body fat is worthy of study in this particular population.

A large number of studies have estimated adiposity with anthropometric measures, with body mass index (BMI) being the most commonly used method. However, the validity of BMI in the study of the relationship between body composition and brain structure and function is somewhat limited and may not fully assess the role of adipose tissue in conferring risk for structural brain alterations^6^. Nevertheless, as BMI is a proximate measure for the degree of adiposity present in the body, it is reasonable to consider the results of other studies with overweight and obese participants in which the BMI and WM relationship was observed^5^. However, the participants of this study are mostly normal-weight, thus different fat compartments were considered as variables of body composition instead of BMI: visceral adipose tissue (VAT) and total fat mass (TFM).

The assessment of WM microstructural changes of the human brain has been widely described from *in vivo* studies using diffusion tensor imaging^7^ (DTI). Many diffusion parameters can be calculated by DTI because of its capacity to evaluate the orientation and magnitude of water diffusion in the whole brain tissue^8^ allowing to observe the relationship between WM integrity and body composition. Concretely, the indicators of WM integrity observed in this research were fractional anisotropy (FA), considered as the directionality of water diffusivity degree, mean diffusivity (MD), an indicator of average molecular motion, and both radial diffusivity (RD) and axial diffusivity (AD), which are MD perpendicular and parallel to axonal fibres respectively.

In this study, we aimed to investigate the relationship of different body fat compartments – (TFM) and (VAT) separately – with WM integrity using a whole-brain approach. To the best of our knowledge, no previous studies have included people whose BMI is normal and their TFM and VAT are low, as may be expected in military pilots.

## Results

### Overall summary

The correlation analysis revealed that higher TFM is associated with higher FA and higher VAT with lower FA, while no significant results were found in MD, RD and AD. Means, standard deviations, minimum and maximum values and 95% confidence intervals for the mean for each variable of the study are displayed in Table 1.

**Table 1:** Summary descriptive statistics for the variables of the study.

Variable	Mean	SD	Minimum	Maximum	95% CI	
					Lower Limit	Upper Limit
Age (years)	37.22	7.90	25	52	33.80	40.63
Height (cm.)	177.61	5.55	167	186	175.21	180.01
Weight (kg.)	80.51	9.26	63	101	76.50	84.51
BMI	25.48	2.49	22	33	24.40	26.55
TFM (kg)	18.32	4.50	10.18	26.36	16.37	20.26
Vat mass (g)	427.65	194.01	238	869	343.75	511.55

Note: SD: Standard Deviation; CI: confidence interval; BMI: body mass index; TFM: total of fat mass, presence of fat mass in the total body mass expressed in kg.; VAT: visceral adipose tissue expressed in g.

### Total fat mass and fractional anisotropy analysis

**Figure 1:**
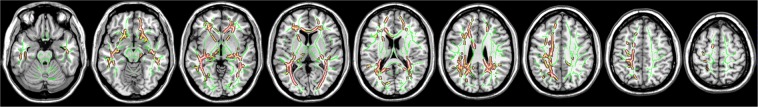
White matter cluster showing a positive correlation between total fat mass and fractional anisotropy. Significant areas are represented in red.

Statistically significant correlations of TFM with white matter fractional anisotropy are reported in Table 2 and depicted in Fig. 1. We observed positive associations of TFM with a set of clusters in the left and right hemispheres, encompassing tracts such as the sagittal stratum, left anterior thalamic radiation, left forceps minor, left inferior fronto-occipital fasciculus, left uncinate fasciculus, left anterior thalamic radiation, right inferior longitudinal fasciculus, right inferior fronto-occipital fasciculus, right splenium of the corpus callosum, forceps major, right inferior longitudinal fasciculus, right inferior fronto-occipital fasciculus (temporal part), right superior longitudinal fasciculus and right anterior thalamic radiation.

**Table 2:** White matter tracts associated with Total Fat Mass.

Tracts	Cluster Index	k	peak p-value	X	Y	Z
Left Sagittal	1	16853	0.006	−43	−22	−14
Right Sagittal stratum	2	5019	0.018	42	25	10
Left Anterior Thalamic radiation	3	514	0.046	−6	−11	−3
Left forceps minor	4	68	0.05	−15	60	−10
Right inferior longitudinal fasciculus	5	30	0.05	17	−75	3
Right splenium of the corpus callosum	6	19	0.05	13	−42	18
Right inferior longitudinal fasciculus	7	14	0.05	46	−28	−14
Right anterior thalamic radiation	8	12	0.05	22	−31	7

Note: k = size in voxels. The listed areas represent the vertex with the maximum difference within the cluster. Coordinates indicate the location of the cluster peak in Montreal Neurological Institute. The *p*-values are corrected for multiple comparisons over both hemispheres using TFCE.

### Visceral adipose tissue and fractional anisotropy analysis

**Figure 2:**
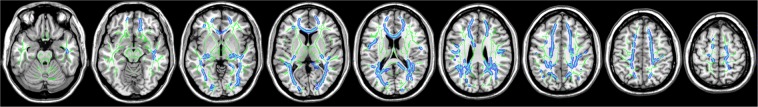
White matter cluster showing a negative correlation between visceral adipose tissue and fractional anisotropy. Significant areas are represented in blue.

**Figure 3:**
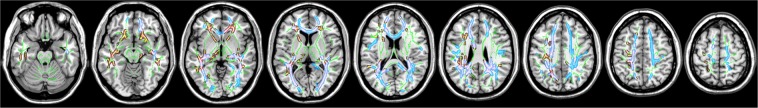
White matter cluster showing both negative and positive correlation between visceral adipose tissue/total fat mass-respectively- and fractional anisotropy. Significant areas are represented in blue for visceral adipose tissue and red for total fat mass.

The correlations between VAT and white matter FA are displayed in Table 3 and Fig. 2. VAT is negatively related to FA in a set of clusters in both hemispheres, involving thalamic radiations, the longitudinal fasciculus, right inferior fronto-occipital fasciculus, sagittal striatum, left inferior fronto-occipital, longitudinal and superior fasciculus; anterior thalamic radiation, the right Genu of the corpus callosum; forceps minor; left posterior thalamic radiation (includes optic radiation); inferior fronto-occipital fasciculus; inferior longitudinal fasciculus; superior longitudinal fasciculus; forceps major, left corticospinal tract; and anterior thalamic radiation Fig. 3.

**Table 3:** White matter tracts associated with visceral adipose tissue.

Tracts	Cluster Index	k	peak p-value	X	Y	Z
Right Posterior Thalamic radiation;	1	8929	0.022	35	−59	1
Sagittal striatum	2	2975	0.046	−41	−37	−8
Right Genu of the corpus callosum	3	2851	0.028	13	32	5
Left Posterior thalamic radiation	4	1437	0.046	31	−67	−1
Left corticospinal tract	5	1228	0.036	−30	−18	50
Left superior longitudinal fasciculus;	6	520	0.048	−51	−4	16
Right inferior longitudinal fasciculus	7	451	0.048	46	−11	−24
Right inferior fronto-occipital fasciculus	8	318	0.048	34	−21	−3
Left Fornix (cres)/Stria terminalis	9	281	0.05	−27	−27	−8
Left inferior fronto-occipital fasciculus	10	128	0.05	−36	−5	−4
Left superior longitudinal fasciculus	11	55	0.05	−31	5	34
Left posterior corona radiata	12	52	0.05	−27	−34	21
Left superior longitudinal	13	45	0.05	−39	−24	29
Left forceps major	14	38	0.05	−11	−50	12

Note: k = size in voxels. The listed areas represent the vertex with the maximum difference within the cluster. Coordinates indicate the location of the cluster peak in Montreal Neurological Institute. The *p*-values are corrected for multiple comparisons over both hemispheres using TFC.

## Discussion

The foremost goal of this study was to examine the possible associations of total fat mass (TFM) and visceral adipose tissue (VAT) with white matter (WM) integrity of subcortical structures on normal-weight individuals (military pilots), through fractional anisotropy (FA), mean diffusivity (MD), radial diffusivity (RD) and axial diffusivity(AD). Most of the previous research has mainly focused this relationship between the total brain volume, grey thickness or white matter, but less on WM integrity. Our results show that the percentages of VAT and TFM correlate negatively and positively with the FA respectively, but not with MD, RD and AD. A possible reason for these not significant results is that, in this population, different fat compartments are not associated with the molecular diffusion rate and myelin presence.

Our results showing that higher presence of VAT is associated with lower FA, are in line with those of other studies aimed at analysing the possible association between body fat mass with brain structure and function principally in obese and elderly people. There is evidence suggesting that adiposity and obesity are associated with altered functional connectivity across different brain regions^9^ and with normal-weight population has been reported that higher VAT is associated with lower functional brain connectivity^5^. Additionally, it seems that several multiple measures of brain microstructure impairs with adiposity^10^. For example, anthropometric and cortical thickness-based measures of visceral fat had a significant inverse association with total brain volume in middle-aged individuals^11^.

Concerning TFM, we observed that in contrary with VAT, is associated with higher FA. To the best of our knowledge, this result is not very present in the literature, perhaps because our participants were physically active and most of them had a BMI of normal-weight. Only one research had a similar result to ours: a cross-sectional study of more than three thousand participants conducted by Croll *et al*.^12^ revealed that higher fat mass is associated with higher FA. Some sample characteristics are quite similar to our study: BMI mean was 27.2 ± 3.9 and they were physically active, although the great differences are that it was composed of elderly people (age mean = 65.9 ± 11.1) and 57.3% were women. Nevertheless, these results support the idea that more studies in which different fat compartments are assessed along with participants with different BMI and not only obese or overweight people are needed.

In overall, these findings are somewhat unexpected, since the associations of TFM and VAT with brain connectivity demonstrate opposite directions. A possible explanation of this result could rely on the different metabolic and endocrine functions associated with different fat compartments. The distribution of body fat is crucial to understanding the adverse effects of obesity^13^. In this respect, brain alterations caused by neurodegenerative, vascular and metabolic disorders have been associated with obesity, particularly with VAT compartment^14^. However, the analysis of subcutaneous fat in participants with acute ischemic stroke did not show correlation with the volume of white matter lesions^13^.

Nevertheless, our findings showed that TFM is positively related to white matter FA in some specific tracts, which may be related to better cognitive function since WM microstructural integrity mediates communications in the brain^15^.

Taking into account the results of the studies described above, and according to our data, it is sound to consider that, in normal-weight population, a certain amount of fat cells may be needed to ensure white matter integrity, while an accumulation on the visceral compartment would hamper the brain connectivity.

It is far to be understood the physiological pathways associated with structural brain changes or connectivity and body fat accumulation. On one hand, there are positive effects associated with adiposity. Several studies have reported that leptin, which is correlated with visceral adiposity, is crucial in the coordination of the excitatory synaptic transmission aroused at hippocampal Schaffer-collateral (SC)-CA1 synapses^16–19^. Leptin receptors are present in various regions of the hippocampus, concretely at hippocampal synapses^20,21^. Moreover, in studies with leptin-insensitive rodents, an impairment in the hippocampal-dependent memory area was observed^22,23^. Thus, it is reasonable to consider that leptin potentially enhance cognitive functions^24^.

Additionally, insulin—which is also correlated with visceral adiposity^25,26^- influences memory processing^27–29^. Most of the insulin receptors are located in areas with an important role in declarative memory formation: the hippocampus and connected limbic brain structures^30–33^. In insulin-administered studies, both intravenous and intranasal administrations have reported an improvement in declarative memory^29,34^. Although it is not clear the mechanisms responsible for this improvement in memory function, Benedict *et al*.^34^ considered that if there is a gradual plastic neuronal change -because of the slow enhancement of memory after insulin inoculation- and that, additionally, improves synaptic plasticity^35,36^, then insulin supports and protects the hippocampal neural connectivity development and, consequently, improves declarative memory.

On the other hand, it is widely accepted that the increase in adipose tissue activity is related to chronic low-grade inflammation^37^ and increasing release of inflammatory cytokines such as leptin, which affects – at least in pathologic conditions^38,39^- the central nervous system due to the activation of microglial cells. Moreover, inflammation could exert deleterious effects on brain integrity through oxidative stress^40^. Another potential mechanism of the negative impact of inflammation on brain integrity is through impairing endothelial function employing a diminished production of nitric oxide^41^. This vasodilator is critical for regulating blood flow in the central nervous system since it helps to maintain resting vascular tone and regulates local flow adjustments in response to hypercapnia demands^42^.

In summary, it seems that adiposity provides both positive and negative effects on brain connectivity. In fact, obesity studies reported that visceral adiposity and BMI –which could be interpreted as the total fat present in the body in overweight and obese populations^5^- are negatively associated with brain connectivity^9–11^, while other studies with normal weight populations such as Croll *et al*.^12^ and ours observed that TFM is positively associated with brain connectivity. According to this information, the authors suggest that the presence of fat may enhance brain connectivity and, probably, overall brain health. However, the accumulation of fat, especially VAT, may hamper brain connectivity. Hence, the presence of fat cells could improve brain health as long as there is no excess, although the mechanisms are not well understood.

Beyond the lack of information on causal directionality, conclusions from the present work are affected by other limitations. The most important is the results reliability, which is limited due to the relatively small sample size in spite of including the entire team of active combat helicopter military pilots in the Spanish Army, although it may be enough to reveal certain patterns considering that the population is rather homogeneous. Future interventions and longitudinal studies should be carried out to assess the relative contribution of each causal fat compartment. Considering that the findings from this study are not fully consistent with the literature, it is necessary to perform further DTI studies aimed at exploring the relationship between TFM/VAT and WM integrity.

In conclusion, DTI results of the present study with combat helicopter military pilots revealed that higher FA is associated with lower VAT and, surprisingly, with higher TFM. The authors suggest that, when there is no excess, fat mass may enhance brain connectivity, while an accumulation of VAT could deteriorate it.

## Material and Methods

### Ethical approval

Recruitment and experimental procedures for this study complied with the Declaration of Helsinki and were approved by the Institutional Review Board (IRB) of the University of Granada, Spain (IRB approval: 850 and 12/CEIH2016). Written informed consent was obtained from each participant prior to the study.

### Participants

Twenty-three military helicopter pilots (M_*age*_ = 36.79; SD = 8.00; M_*BMI*_ = 25.48; SD = 2.49) from the Spanish Army Airmobile Force (FAMET), serving at the Almagro airbase, Ciudad Real (Spain), took part in the study. The sample size was limited by participants’ availability. All pilots were members of the Attack Helicopter Battalion BHELA I. Fifteen were qualified to fly the EC665 Tigre attack helicopter, constituting the entire active team of Spanish Tigre pilots, and eight out of the twenty-three flew the Messerschmitt-Bölkow-Blohm Bo 105 helicopter. All volunteers were on flight status, had a recent verification of good health and were free of medication. Academic training and military ranking were diversified, between the level of brigadier and lieutenant colonel.

### Variables and instruments

#### Analysis of cerebral connectivity

Regarding the analysis of cerebral connectivity, T1-weighted and DTI volumes were acquired using 32-channel Siemens 3 T Magneton TRIO system located at the Mind, Brain and Behaviour Research Centre (CIMCYC) at the University of Granada. The T1 MPRAGE parameters were: TR = 1900 ms; and TE = 2.52 ms; FA = 9°). A total of 176 slices of a thickness of 1 mm of the whole brain were obtained (voxel size = 1 × 1 × 1 mm^3^, FOV = 256 mm, 256 × 256 data acquisition matrix).

Diffusion images were acquired on the same scanner using a 2D EPI diffusion sequence, with the following parameters: TE = 90 ms and TR = 3300 ms. A DTI diffusion plan was used, and a total of 30 sampling directions, repeated 3 times. In addition, 3 b0 volumes were obtained. The value of b was 1000 s/mm^2^. The in-plane resolution was 1,797 mm. The thickness of the slice was 5.2 mm. The DTI volumes were analysed with the FDT program^43^, a part of the library FMRIB, v5.5^44^. The images were corrected by a brain mask created from the b0 images, and the FA maps were obtained by applying dtifit to the masked volumes. The analysis was carried out using the tract-based spatial statistical package (TBSS). The FA maps were recorded by the standard FA template FSL in MNI (Montreal Neurological Institute) space using FNIRT. The mean volume FA was used to skeletonize the individual normalised FA maps. This skeletonization is assumed to be the centre of all sections following a given path. The individual maps of the FA were projected to these skeletons and voxel-wise analysed using Threshold-free Cluster Enhancement (TFCE, a part of the FMNI library).

#### Body composition

Dual-energy X-ray Absorptiometry (DXA) fan-beam technology (Hologic QDR 4500 A, Hologic) was used for the measurement of BMI, TFM and VAT. A standardized procedure for patient positioning and utilization of the QDR software was used. The DXA 4500 A scans were analysed with the software version 8.21.

### Statistical analysis

Data summaries were computed for the whole sample. Shapiro-Wilk’s tests indicated normality for the variables of interest. For each correlation analysis (TFM and VAT), a total of 2,500 random extractions were given to obtain an empirical distribution of the correlation coefficient. A TFCE corrected for a multiple comparisons p-value of 0.05 was used.

## Data Availability

The corresponding author had full access to all the data in the study and all authors shared final responsibility for the decision to submit for publication. The database is available without restriction in the Open Science Framework (OSF) website, https://osf.io/kq8ph/.
